# Estimating the geographic distribution of human Tanapox and potential reservoirs using ecological niche modeling

**DOI:** 10.1186/1476-072X-13-34

**Published:** 2014-09-25

**Authors:** Benjamin P Monroe, Yoshinori J Nakazawa, Mary G Reynolds, Darin S Carroll

**Affiliations:** Division of High-Consequence Pathogens and Pathology, Centers for Disease Control and Prevention, 1600 Clifton Road NE, Mailstop A-30, Atlanta, GA 30333 USA

**Keywords:** Poxvirus, Environmental niche modeling, Disease reservoir

## Abstract

**Background:**

Tanapox virus is a zoonotic infection that causes mild febrile illness and one to several nodular skin lesions. The disease is endemic in parts of Africa. The principal reservoir for the virus that causes Tanapox is unknown, but has been hypothesized to be a non-human primate. This study employs ecological niche modeling (ENM) to determine areas of tropical Africa suitable for the occurrence of human Tanapox and a list of hypothetical reservoirs. The resultant niche model will be a useful tool to guide medical surveillance activities in the region.

**Methods:**

This study uses the Desktop GARP software to predict regions where human Tanapox might be expected to occur based on historical human case locations and environmental data. Additional modeling of primate species, using occurrence data from museum records was performed to determine suitable disease reservoirs.

**Results:**

The final ENM predicts a potential distribution of Tanapox over much of equatorial Africa, exceeding the borders of Kenya and Democratic Republic of Congo (DRC) where it has been historically reported. Five genera of non-human primates were found to be potential reservoir taxa.

**Conclusions:**

Validity testing suggests the model created here is robust (p < 0.04). Several genera of primates were identified as having ENMs overlapping with that of Tanapox and are suggested as potential reservoirs, mainly members of the Genus *Cercopithecus*. The ENM modeling technique has several limitations and results should be interpreted with caution. This study may increase knowledge and engage further research in this neglected disease.

**Electronic supplementary material:**

The online version of this article (doi:10.1186/1476-072X-13-34) contains supplementary material, which is available to authorized users.

## Background

Tanapox virus, a member of the genus *Yatapoxvirus*, is a zoonotic virus that causes mild febrile illness and nodular skin lesions in humans and non-human primates [1, 2]. The virus is sufficiently distinct from the related orthopoxviruses –including variola, and monkeypox— that traditional smallpox vaccination offers no cross-reactive immunity to Tanapox [3]. The disease was first observed among humans living in the flood plain of Kenya’s Tana River in 1957 [4]. Additional instances of human disease have been reported in animal handlers at primate facilities in the United States and among international travelers who visited tropical Africa [5–7]. As well, Tanapox was reported extensively from the Democaratic Republic of Congo (DRC) during the period of heightened surveillance post-smallpox eradication.

Clinical disease in humans begins with a febrile prodrome (fever 38-39°C) that can include headache, backache, prostration, and lymphadenopathy. This period lasts for 1–2 days and is followed by lesion development. Lesions evolve to become firm, deep-seated, elevated nodules reaching 20 mm in diameter, which frequently break or become ulcerated, often leading to secondary infections. A patient may have only a single nodule, presumably at the site of virus entry into the skin, or Tanapox lesions can occur in crops of 2–10 [8]. Lesions typically resolve after 6 to 8 weeks with scarring often present [9]. The disease is rarely reported and may be confused with scabies, insect bites, or tropical ulcers [2]. An additional factor for low reporting may could be that patients with limited resources may also avoid seeking medical care as the disease has a relatively benign course.

Over a 40-year period from 1957 to 2003 human Tanapox disease has been sporadically identified across the breadth of Africa; spanning more than 6,000 kilometers from Sierra Leone to Tanzania [4–8]. The area covered includes a range of climatic aspects and several distinct ecological regions [10]. Tanapox is a zoonosis, with non-human primates highlighted as prospective reservoirs [11] (Additional file 1). Studies conducted in Kenya in 1971, after a large outbreak of Tanapox, revealed an association between the prevalence and activity patterns of biting arthropods and Tanapox occurrence leading to the suggestion that arthropods may play a role in outbreaks of human disease [12]. It is conceivable that biting insects could play a role in transmitting the virus mechanically, by moving the virus passively via mouthparts from one person to another.

The sporadic nature of human Tanapox renders traditional prospective epidemiology studies for determination of risk impracticable. Human Tanapox is not a reportable disease in endemic regions, so traditional surveillance systems will not reliably enumerate cases. Medical staff in these areas may be insufficiently trained to recognize the symptoms of Tanapox, and modern diagnostic testing is generally not available. Novel methods for determining disease risk are needed. Here we focus on identifying presumptive zoonotic risks by searching for ecologic commonalities between where Tanapox occurs and the likely reservoir species that live there.

Due to the high diversity of mammalian taxa which interact with humans in these regions it can be very difficult to determine particular taxa involved in disease transmission. For the purpose of this study we have assumed that primates are the likely reservoir based on laboratory findings that indicated only primates show evidence of productive infection after being experimentally inoculated [4]. Other taxa examined, which did not demonstrate Tanapox virus-induced illness, include calves, lambs, pigs, goats, rabbits, guinea-pigs, and mice [4]. Many species of primates exist in the locations where human disease occurs. Human-primate interactions are also common in these locations, where primates may be sold, kept as pets, or serve as agricultural pests [13]. The ecological niche modeling (ENM) technique known as the Genetic Algorithm for Rule-Set Production (GARP) relates ecological information from the landscape of disease to create a set of rules that define factors associated with disease presence [14]. The final result, an (ENM) can be rendered on a map depicting the potential geographic distribution for the pathogen. This study uses the Desktop version of GARP to model the predicted presence of human Tanapox from case locations available in the literature and records from Centers for Disease Control and Prevention (CDC) and World Health Organization (WHO) surveillance activities. In addition, we evaluate a list of potential primate reservoirs to determine which have ranges that coincide with human Tanapox.

## Results

### Ecological niche modeling

**Figure 1:**
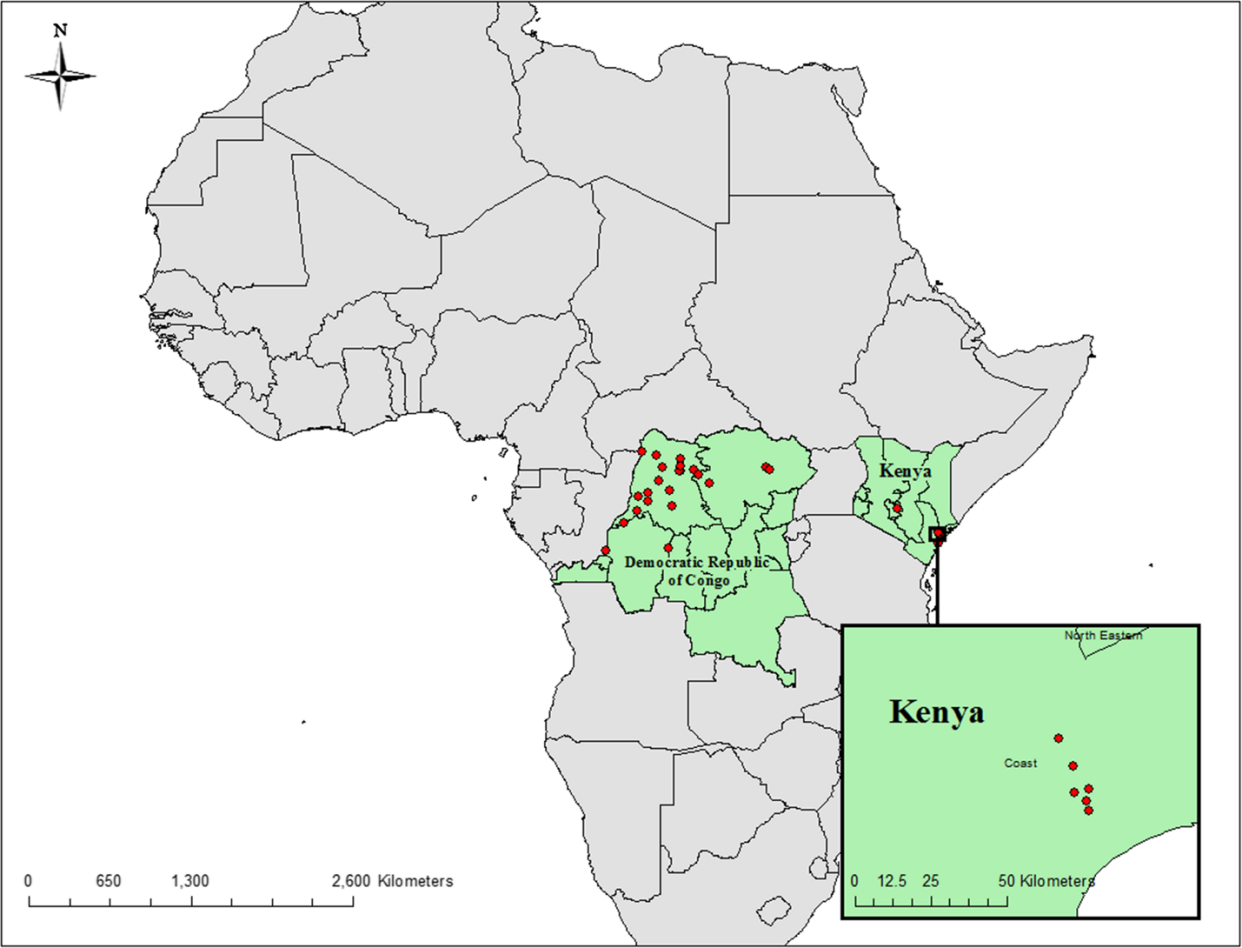
**Reported locations of Tanapox cases, 1971–1986.** Cases of human Tanapox were identified from World Health Organization smallpox eradication records in the Democratic Republic of Congo and from published reports from Kenya [12].

**Figure 2:**
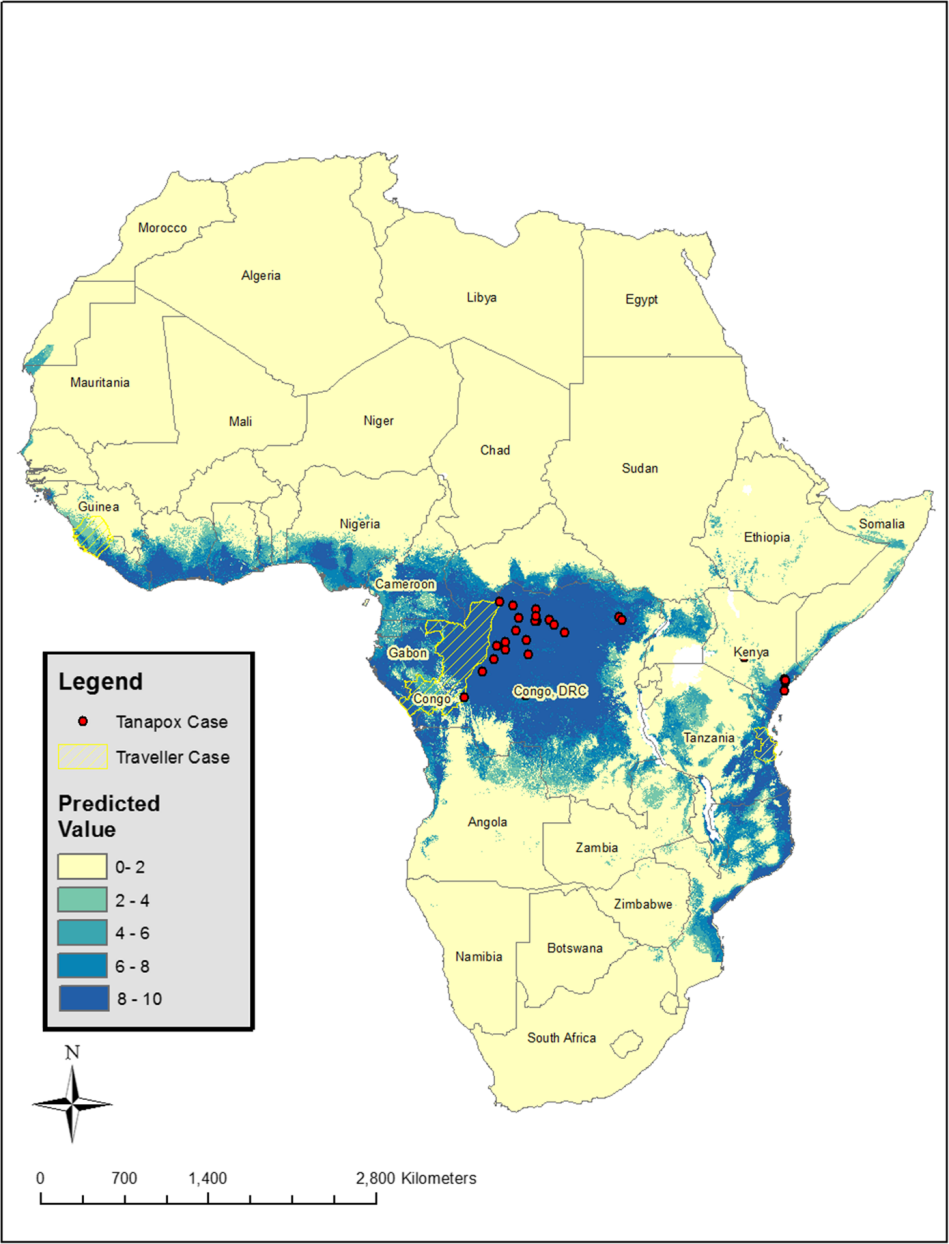
**Ecological niche model of Tanapox.** Overall predicted distribution of human Tanapox from GARP modeling. Darker areas represent higher model agreement; locations used for modeling are shown as red triangles. Areas highlighted in yellow represent locations visited by international travelers confirmed to have Tanapox (not used in modeling).

Niche models were generated according to the methods outlined by Stockwell [14] and are described in detail below. Due to incomplete geographic reference information, only 30 unique occurrence locations (8 from Kenya and 22 from DRC) from 214 enumerated human cases could be assigned coordinates accurate to 0.01 decimal degrees (Figure 1). The final 10-subset model predicted the presence of human Tanapox at 28 (93.3%) case locations The final model predicts a potential distribution of Tanapox over much of equatorial Africa with the areas with the highest predicted values including a majority of forested Central Africa (DRC, Republic of Congo, Gabon Guinea and Cameroon), coastal East Africa (Kenya, Tanzania, and Mozambique), and West Africa (Nigeria, Togo, Ghana, Ivory Coast, Liberia, and Sierra Leone) (Figure 2). Isolated areas of Guinea-Bissau, Northern Uganda, and Malawi (surrounding Lake Malawi) were also potentially suitable for human Tanapox. Additional modeling iterations, each performed after eliminating a single environmental layer (jackknife analysis), revealed that the layers with most influence on model projections were maximum temperature, minimum temperature, and precipitation (I = 0.728, 0.738, and 0.741 respectively) (Table 1).

**Table 1:** **Summary of environmental characteristics used in creation of Tanapox ENM**

Layer	Hellinger’s I
Maximum temperature	0.728798459
Precipitation	0.738374454
Minimum temperature	0.741624095
Slope	0.743987125
Land Cover	0.756236193
Elevation	0.756455174
Flow Direction	0.760079558
Temperature Range	0.767203506
Mean Temperature	0.770258725
Compound Topographic Index	0.774950877

Hellinger’s I represents niche overlap with values between 1 (niches identical) and 0 (no relationship). Lower values represent more influence on determination of final model.

### Model robustness

The ENMs created for Tanapox with Desktop GARP reached the predetermined convergence threshold of 0.01 prior to the maximum of 1,000 iterations. This study employed a previously described partial ROC analysis to compare the performance of the model at *biologically plausible* thresholds of sensitivity and specificity [15]. An AUC ratio of 1.35 (p > 0.04, null value =1) was calculated from the partial ROC analysis; indicating that the model predicts occurrence points better than would occur at random.

Tanapox occurrence points were also divided into four quadrants. Models created from points in diagonally adjacent quadrants were used to predict the locations in the reciprocal quadrants. The ENM trained by points in the northwest-southeast quadrants, predicted all twelve points in the opposite quadrants to be suitable for Tanapox. In the following model trained with northeast-southwest quadrants, 15 of 17 points (88.2%) were predicted as suitable for Tanapox.

### Reservoir analysis

**Figure 3:**
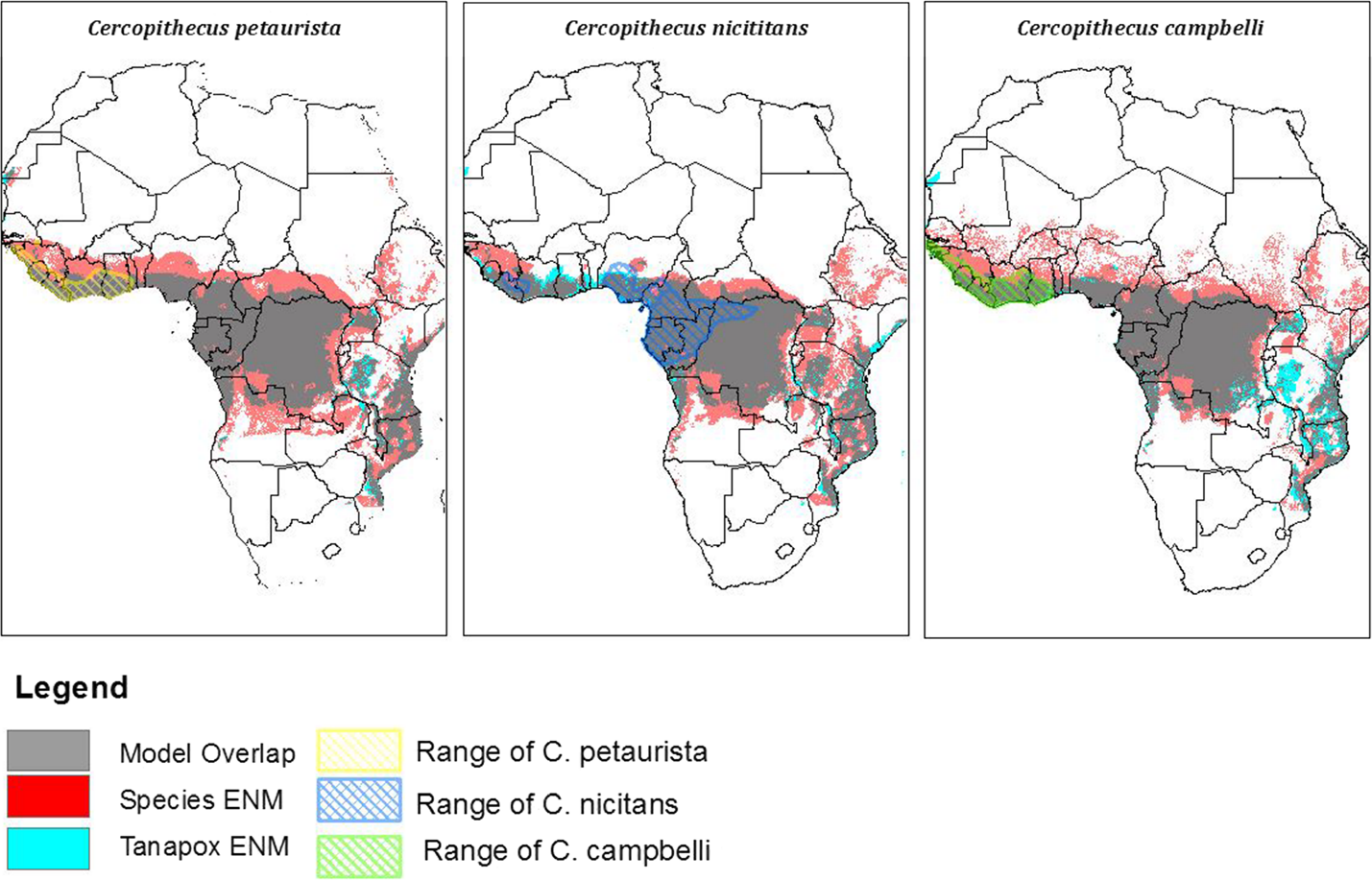
**Comparison of Tanapox and Primate ENMs.** Displayed are the overlap of ENM’s for Tanapox and three hypothesized primate reservoirs (*C. petaurista, C. nicitans, C. campbelli*). Areas of niche overlap are displayed in gray. The current species distribution for each primate was retrieved from the International Union for the Conservation of Nature (IUCN, http://www.iucn.org/) and is shown in the crosshatched area.

A review of *Wilson and Reeder’s Mammal Species of the World, 3rd edition* found eighteen genera of primates to have distributions that include countries from which historical cases of Tanapox have been reported (Republic of Congo, DRC, Tanzania, Kenya, and Sierra Leone) [16] (Table 2). The geographic ranges of these primates were compared on the basis of range overlap to the distribution of human Tanapox in Africa. Of these genera, 13 were excluded from further consideration based on extremely isolated or broad primate ranges compared to the Tanapox ENM based on human disease occurrences. The five remaining primate genera were *Galago, Cercopithecus, Cercocebus, Colobus*, and *Pan*. ENMs for species within each of the highlighted genera were created with point data derived from the latitude and longitude of collection from museum specimens (http://data.gbif.org/welcome.htm). Sufficient data were available to create ENMs of 15 species within the selected genera. The species with the highest agreement are shown in Table 3. Several *Cercopithecus* species (*C. petaurista, C. nicitans, C. campbelli, C.ascanius*) and *Cercobus albigena* had a potential range with a high degree of niche overlap. The potential distributions for these species often overlap geographically in West and Central Africa with some addition range area in Uganda, Kenya Zambia, and Angola (Figure 3).

**Table 2:** **Summary of primates with ranges including countries where Tanapox has been described**

Genus	Common name	Range	No. of species
Infraorder Lorisiformes			
*Acrtocebus*	Golden Potto	Central Africa	2
*Perodicticus*	Potto	West and Central Africa; Kenya	1
*Euoticus*	Bushbabies	Nigeria; Cameroon; Gabon; ROC	2
*Galago*	Galagos	Throughout Sub-Saharan africa	14
*Otolumur*	Greater Bushbabies	Kenya; Tanzania; Rwanda; Angola	3
*Family Cercopithecidae, subfamily cercopithecinae*			
*Erythrocebus*	Patas Monkey	West Africa to Ethiopia, Kenya, and Tanzania	1
*Chlorocebus*	Savanah Guenons	Throughout Africa	6
*Cercopithecus*	Guenons	Throughout Africa	25
*Miopithecus*	Talapoin	Cameroon; Gabon; Angola; DRC	2
*Allenopithecus*	Allen’s Monkey	DRC	1
*Cercocebus*	Mangabeys	Central and West Africa; Kenya; Tanzania	6
*Lophocebus*	Black Mangabeys	West and Central Africa; Uganda; Burundi; Angola	3
*Papio*	Baboons	Throughout Africa	5
*Mandrillus*	Mandrill	Nigeria; Cameroon; Equatorial Guinea	2
*Family Cercopithecidae, Subfamily Colobinae*			
*Colobus*	Colobus Monkeys	Throughout Sub-Saharan Africa	5
*Piliocolobus*	Red Colobus Monkey	West, Central, and East Africa	9
*Family Pongidae*			
*Gorilla*	Gorilla	Central and West Africa	2
*Pan*	Chimpanzees	Central and West Africa; W Tanzania	2

The primate genera with ranges including any part of Democratic Republic of Congo, Republic of Congo, Tanzania, Kenya, Sierra Leone [16].

**Table 3:** **Comparison of selected primate and human Tanapox ENMs**

Family	Genus	Species	Ecological models		
			Niche overlap	Rank	Points modeled
Lorsidae					
	***Galago***				
		*G. demidoff*	0.648	9	28
Cercopithecidae					
	***Cercopithecus***				
		*C. petaurista*	*0.729*	*1*	*25*
		*C. nictitans*	*0.712*	*2*	*16*
		*C. campbelli*	*0.692*	*3*	*22*
		*C. ascanius*	*0.669*	*5*	*13*
		*C. mitis*	*0.616*	*10*	*37*
		*C. aethiops*	*0.577*	*12*	*63*
		*C. diana*	*0.549*	*13*	*10*
		*C. lowei*	*0.517*	*14*	*12*
	***Cercocebus***				
		*C. albigena*	0.670	4	14
		*C. atys*	0.658	7	13
		*C. torquatus*	0.613	11	11
	***Colobus***				
		*C. Guereza*	0.493	15	37
		*C. polykomos*	0.667	6	15
Pongidae					
	***Pan***				
		*P. troglodytes*	*0.648*	*8*	*26*

The 15 primate species with the highest degree of niche overlap, and the number of points used in creation of the model.

## Discussion

The ENM of human Tanapox predicts a potential distribution over much of tropical Africa. Areas predicted in 8 or more models included the forested areas of the Congo basin and West Africa, coastal areas of East Africa, and areas surrounding The African Great Lakes (Lake Albert, Lake Tanganyika and Lake Malawi). While predicted areas in DRC and coastal Kenya would be expected based on input data, the remaining areas indicate potential undescribed presence. In general the model predicts that Tanapox could be found in areas of the continent with high temperatures and rainfall.

Three cases of Tanapox that were reported in travelers returning from Africa were not included in construction of the model because the precise geographic location of the presumed exposure is not known [5–7]. The general locations visited by the travelers included, southeast Republic of Congo, Bagamoyo district of Tanzania, and Sierra Leone. All three of these areas were broadly predicted areas of Tanapox presence (Figure 2).

Among the advantages of using an ENM approach to identify geographic locales with the potential to support pathogen transmission, is the additional information that can be obtained from parsing out which ecologic features most influence the model’s predictive capacity. The identification of temperature (maximum and minimum) and precipitation as the environmental variables with the most influence may prove useful in determining the fauna responsible for disease transmission. The initial reports of Tanapox indicate flooding coinciding with an abundance of mosquito activity prior to the 1957 outbreak [17]. This has led to a theory that insects might be involved in transmission. It has additionally been suggested that Tanapox nodules are more commonly found on the extremities because those areas are often not covered by clothing and susceptible to biting insects [8]. Temperature and precipitation are often linked to insect development, and their influence here may support the theory of arthropod transmission [18].

Several of the primate ENMs used in this study have a high degree of overlap with the ENM created from human Tanapox. In general, areas of West, Central, and Coastal East Africa appear to have environmental aspects suitable for both the highlighted *Cercopithecus* and *Cercocebus* genera and human Tanapox. No single species, however, was able to be highlighted as a reservoir. Additionally, the primates with the highest degree of spatial overlap have ranges restricted to West and Central Africa, and therefore are unable to cause human disease in Kenya. This may indicate that the ENMs for both Tanapox and the hypothesized primates are simply ‘finding the forests’ or that a pool of primate species may host the virus. A scenario where overlapping primate ranges with similar environmental characteristics like rainfall and temperature may allow the pathogen to spread from one side of the continent to the other.

Primates have been implicated in Tanapox disease based on both infections at animal facilities and observations of primates in areas where human disease occurred. *Cercopithecus* and *Cercocebus* monkeys were noted as present in areas with frequent Tanapox infections in Kenya and have been found to have neutralizing antibodies to the Tanapox virus in laboratories [4, 19]. The genus *Cercopithicus* contains the most widespread primates in Africa, and are often found in areas that have undergone environmental change associated with human activity including clearing forest for roads, logging, and crop cultivation [20]. *Cercopithicus nictitans* are known to disperse throughout narrow riverine areas, and have a distribution that closely mirrors river basins. *Cercocebus* mangabeys are noted to be mainly terrestrial and often inhabit areas close to rivers; areas often mentioned in Tanapox outbreaks in Kenya. These primates inhabit the habitat surrounding human settlements and are often found raiding farms, hunted for meat, or kept as pets [20].

Omnivorous primates known to subsist on fruits and insects in undisturbed forest, will raid crops in forest fragments created by encroaching agriculture. Periods of heightened crop raiding closely mirror peak agriculture production following seasonal rains (primates may prefer cultivated maize to wild fruits) [21]. Humans in these areas have additional exposure to primates while entering forest fragments for daily activities (collecting firewood or water) [21]. The red-tailed monkey, *Cercopithecus ascanius*, show a proclivity for crop raiding and residents in communities bordering Kibale National Park in Uganda were more likely to harbor bacteria similar to those found in *C. ascanius*, than other forest primate species [22]. Primates that live in close proximity to humans and have behavior that would facilitate human-primate interactions may similarly facilitate Tanapox transmission and warrant future investigation.

This type of modeling has several limitations, and caution is necessary during interpretation. This analysis is based on the location of human cases, which can often be improperly reported and serve only as a proxy to the complex ecological interactions necessary to model the actual pathogen [23]. The coarse spatial resolution (1 km) of the environmental data used in modeling is also a limiting factor in the spatial resolution in the final map. The ENMs for primates relied on data available from online databases and models could only be constructed for species with sufficient data. While results are given at the species level, they should be interpreted in a broader context. GARP models predict areas of potentially suitable habitat or potential niche, not necessarily the current range or realized niche of a species [24]. This may be especially so with primates, where complex social and behavioral characteristics may govern habitat selection. This method of investigation is not meant to serve as a replacement for biologic or epidemiologic studies, but instead serves as a way to renew research interests in a long-neglected disease.

## Conclusions

The creation of an ENM for Tanapox predicts a range that includes areas of historical presence, recent anecdotal reports from travellers, and an expanded area of favourable ecological habitat. Also presented here is a short list of primates that have ecological ranges concordant with human Tanapox and may serve as suitable disease reservoirs. In the fifty years since the disease was first isolated, only a handful of articles have been published on the subject. This study aimed to use ecological modeling techniques to provide additional evidence to an often-neglected disease burden in humans. The results of this research are a step to improving the ability of healthcare workers in resource-limited areas of Africa to diagnose and enhance surveillance for cases of Tanapox. It is hoped this research can be used as a guide to maximize future research by limiting the time and effort spent sampling in areas with low environmental suitability and highlighting a short list of pirmates that might serve as sylvatic hosts or reservoirs of this diseae.

## Methods

### Tanapox occurrence data

Tanapox occurrence data from sub-Saharan Africa were compiled from two distinct sources. i) A 1971 serology survey of the Tana River valley in Kenya found 31 individuals at 10 locations to have neutralizing antibodies positive for Tanapox, an indication of infection in the previous 12 months [12]. ii) A line by line search of legacy surveillance notebooks from the smallpox eradication era from 1974–1986 yielded 206 cases of Tanapox in DRC (recorded as Zaire), confirmed to have enveloped virus by electron microscopy (Additional file 2: Table S2). Locations were geo-referenced using the geographic names database (GNS, http://earth-info.nga.mil/gns/html/index.html) from the National Geospatial-Intelligence Agency. Due to the complicated geography of the area and incomplete gazetteer databases, only 214 (90.2%) of the records at 30 distinct locations (8 from Kenya and 22 from DRC) could be assigned specific coordinates (Additional file 3).

Case localities may not account for the actual place of infection if persons traveled in order to visit a health facility or if the location was not georeferened properly. To account for the uncertainty associated with acquiring disease, 10 randomly generated points within a circular buffer surrounding each case were selected for modeling as described by Nakazawa et al. [25]. Cases from DRC and Kenya were assigned 20 km and 50 km polygons respectively to account for movement of persons before the case was reported (Cases in Kenya were given a larger polygon to account for a longer time span before a case was identified). No person-to-person transmission has been reported for Tanapox so each of these cases can be considered as an independent wildlife-to-human infection, allowing polygons to be weighted for multiple cases at a single location. The modeling procedure was performed with 10 unique datasets that contained 214 randomly generated points representing each reported case selected from within 30 distinct polygons representing the distinct location where cases were reported. The final model was created by aggregating the 10 binary (1 = presence, 0 = absence) output models on a pixel by pixel basis. Data describing a suite of 10 environmental characteristics were available for 29 locations (one location was masked by water).

### Environmental data

Environmental data used to create the ENMs included 10 distinct geographic layers from three main sources (1) Climatic data representing the period 1950–2000, including layers representing annual mean temperature, maximum temperature, minimum temperature, diurnal temperature range, and precipitation obtained from the WorldClim data set [26]. (2) Topographical and hydrological data representing elevation, slope, aspect ratio, compound topographic index (water pooling), were derived from the United States Geological Survey’s Hydro-1 K dataset (http://eros.usgs.gov/#/Find_Data/Products_and_Data_Available/gtopo30/hydro/africa). (3) Categorized landcover data for Africa available from the International Steering Committee for Global Mapping (ISCGM, http://211.19.49.27/gmd/download/glcnmo.html). All analyses were carried out using a spatial resolution of 0.01° (approximately 100 m^2^ pixels) with ArcGIS version 9.3 (ESRI, Redlands, CA).

### Ecological Niche Modeling of Tanapox

ENMs were built using Desktop GARP (http://www.nhm.ku.edu/desktopgarp/). GARP uses an evolutionary computing method applied to a random set or rules (bioclimatic rules, range rules, logistic regression) that are ‘evolved’ in an iterative process to maximize the predictive ability of the model and has been described thoroughly [14, 27]. For this investigation, each of the 10 occurrence datasets was run 20 times, resulting in 200 total GARP models. Within each dataset, 19 of the 20 models were filtered out using the “best subsets selection parameters” available in the software (‘soft’ omission threshold of 10% and 50% commission threshold) [24]. The selected model from each of the 10 datasets was combined to show agreement between runs and to produce an overall prediction map. The number of times out of 10 that a specific pixel was predicted was symbolized with color gradations (darker representing higher model agreement) (Figure 2) of the map representing continental Africa.

### Model robustness

A method to determine the ability of an ENM to predict a poxvirus into an area where no occurrence data are represented has been described as the ‘quadrant test’ [28]. Occurrence points were divided into four quadrants based on their position above or below longitude and latitude of the central point (22.758 E, 1.893 N). ENMs were trained with points from two oblique quadrants (e.g. northwest and southeast) with points in the remaining quadrants used to independently test model predictability. The reciprocal process was repeated for the remaining quadrants. A final method used to test model quality was the modified receiver operating characteristic curve (ROC) outlined by Peterson et al. [15].

### Principal environmental factors

In order to explore the effect of each environmental characteristic to the overall model jackknife procedure was performed similar to what has been published elsewherere [29]. Niche identity tests were performed on the Tanapox ENM to compare each of the jackknifed models to the map including all environmental layers. It was assumed that models missing key environmental characteristics would have the highest divergence from the human Tanapox model. The suite of models was ranked in ascending order with the ENM tools software package (retrieved from http://enmtools.blogspot.com/search?q=cite).

### Reservoir analysis

Using Wilson and Reeder’s *Mammal Species of the World*, 3^rd^ edition, a primate genus was recorded if a portion of its known range occurred in a country where previous cases of Tanapox had occurred (DRC, Republic of Congo, Tanzania, Kenya, Sierra Leone). This included 18 primate genera and 91 species. The estimated ranges for these primates were extracted from the International Union for Conservation of Nature (IUCN) list of terrestrial mammals (http://www.iucnredlist.org/technical-documents/spatial-data). Using the map comparison kit software (http://www.riks.nl/mck/), overlap between the primate ranges and the human Tanapox ENM was determined by Fuzzy Kappa. The five genera with the highest agreement were selected for further analysis. Within these genera, occurrence points were available for 15 species from the Global Biodiversity Information Facility (http://data.gbif.org/occurrences/). Species with over 25 occurrence points were modelled 50% training-50% testing. Species with less that had less than 25 points were 100% training. GARP models were developed for each species independently using the same environmental layers for the human Tanapox ENM. Species distributions were compared to human Tanapox on the basis of niche overlap from Hellenger distance estimation in the ENM Tools software package.

## Electronic supplementary material

Additional file 1: **Summary of primates with published links to Tanapox.** Included are anecdotal reports from the literature of primates possibly associated with Tanapox. (DOCX 16 KB)

Additional file 2: Table S2: Coordinates for Tanapox case locations. (DOCX 26 KB)

Additional file 3: **Coordinates for primate species records used in ENM modeling.** Data from http://www.gbif.org was used to create ENMs for the 15 primate species. (JPEG 234 KB)
