# Supplementary material for: Estimating the geographic distribution of human Tanapox and potential reservoirs using ecological niche modeling
Source: Int J Health Geogr. 2014 Sep 25;13:34. doi: 10.1186/1476-072X-13-34 (PMC4189193; doi:10.1186/1476-072X-13-34)
Supplement: Supplementary file 1 — Additional file 1: Summary of primates with published links to Tanapox. Included are anecdotal reports from the literature of primates possibly associated with Tanapox. (DOCX 16 KB) [file 12942_2014_602_MOESM1_ESM.docx]

**Additional files**

**Additional file 1 – Summary of primates with published links to Tanapox**

Included are anecdotal reports from the literature of primates possibly associated with Tanapox.

| Scientific Name | Common Name | Range | Relationship to tanapox | Reference |
| --- | --- | --- | --- | --- |
| *Procolobus rufomitratus* /*pilocolobus* | Red Colobus Monkey | Uganda; Rwanda; Burundi; Tanzania; Kenya | Serologic evidence of poxvirus infection, habitat near Tana River | Goldberg, 2008 |
| *Papio anubis* | baboon | Mali to Ethiopia; Kenya; Tanzania | yaba-like condition found | Whittaker, 1985 |
| *Cercopithecus aethiops* / *Chlorocebus sabaeus* | African green monkey | Senegal to Ghana | reported to be natural host | Downie, 1974 |
| *Erythrocebus patas* | patas monkey | W. Africa to Ethiopia; Kenya; Tanzania | tanapox neutralizing antibody present | Downie, 1974 |
| *Pan troglodytes* | chimpanzee | W Africa; Cameroon; Gabon; ROC; Uganda; Tanzania; DRC; CAR | tanapox neutralizing antibody present | Downie, 1974 |
| *Colobus abyssinicus* / *Colobus angolensis* | colobus monkey | Angola; DRC;Rwanda; Durundi; Zamibia; Kenya; Tanzania | tanapox neutralizing antibody present | Downie, 1974 |
| *Chlorocebus pygerythrus* | vervet | Ethiopia; Somalia; Zambia; South Africa | laboratory testing | Downie, 1971 |
| *Papio doguera* / *cynocephalus* | dog face baboon | Kenya | present in locality of outbreak | Downie, 1971 |
| *Cercopithecus mitis* | blue monkey | DRC; ROC; Angola; Zambia; Kenya; Tanzania | present in locality of outbreak | Downie, 1971 |
| *Cercocebus galeritus* | Mangabey | Kenya | present in locality of outbreak | Downie, 1971 |
