# Supplementary material for: Estimating the geographic distribution of human Tanapox and potential reservoirs using ecological niche modeling
Source: Int J Health Geogr. 2014 Sep 25;13:34. doi: 10.1186/1476-072X-13-34 (PMC4189193; doi:10.1186/1476-072X-13-34)
Supplement: Supplementary file 2 — Additional file 2: Table S2: Coordinates for Tanapox case locations. (DOCX 26 KB) [file 12942_2014_602_MOESM2_ESM.docx]

**Additional file 2- Coordinates for Tanapox case locations**

| Source | Country | Location | Count | longitude (DD) | latitude (DD) |
| --- | --- | --- | --- | --- | --- |
| Downie 1973 | Kenya | Ngao | 1 | 40.209167 | -2.421667 |
| Downie 1973 | Kenya | golbanti | 1 | 40.202500 | -2.456667 |
| Downie 1973 | Kenya | Gumba | 1 | 37.133333 | -0.600000 |
| Downie 1973 | Kenya | Oda | 1 | 40.207500 | -2.483889 |
| Downie 1973 | Kenya | Garsen | 1 | 40.120000 | -2.269722 |
| Downie 1973 | Kenya | Mambrui | 1 | 40.150000 | -3.116667 |
| Downie 1973 | Kenya | Tarasa | 1 | 40.166667 | -2.433333 |
| Downie 1973 | Kenya | Kibusu | 1 | 40.163611 | -2.352222 |
| CDC/WHO | DRC | lisala | 171 | 21.516670 | 2.150000 |
| CDC/WHO | DRC | Camp Akula | 4 | 20.200000 | 2.366667 |
| CDC/WHO | DRC | Umangi | 2 | 21.400000 | 2.116667 |
| CDC/WHO | DRC | Boso-Kuluki | 4 | 19.950000 | 1.400000 |
| CDC/WHO | DRC | Bangala | 1 | 19.150000 | -0.016670 |
| CDC/WHO | DRC | Ngauda yanza | 1 | 20.683300 | -3.466670 |
| CDC/WHO | DRC | Mbuni | 2 | 18.346920 | -0.763170 |
| CDC/WHO | DRC | Bumba | 5 | 22.466670 | 2.183330 |
| CDC/WHO | DRC | Ngombe-doko | 1 | 27.683330 | 2.400000 |
| CDC/WHO | DRC | Mambenga | 1 | 27.933330 | 2.183333 |
| CDC/WHO | DRC | Boende | 3 | 20.878949 | -0.282416 |
| CDC/WHO | DRC | Boso-likulu | 1 | 19.200000 | 0.550000 |
| CDC/WHO | DRC | Mondongo | 1 | 21.483330 | 2.966667 |
| CDC/WHO | DRC | Bobala | 1 | 21.500000 | 2.483333 |
| CDC/WHO | DRC | Mongo | 1 | 18.716670 | 3.533333 |
| CDC/WHO | DRC | Basoko | 1 | 23.600000 | 1.233333 |
| CDC/WHO | DRC | Bapota | 1 | 20.733330 | 0.716667 |
| CDC/WHO | DRC | Yamonomo | 1 | 22.800000 | 1.833333 |
| CDC/WHO | DRC | Gemena | 1 | 19.766670 | 3.250000 |
| CDC/WHO | DRC | Kaba | 3 | 16.100000 | -3.583333 |
| CDC/WHO | DRC | Monzambi | 1 | 18.500000 | 0.283333 |
| CDC/WHO | DRC | Wanga | 1 | 17.450000 | -1.650000 |
