# Supplementary figures and images for: Estimating the geographic distribution of human Tanapox and potential reservoirs using ecological niche modeling
Source: Int J Health Geogr. 2014 Sep 25;13:34. doi: 10.1186/1476-072X-13-34 (PMC4189193; doi:10.1186/1476-072X-13-34)

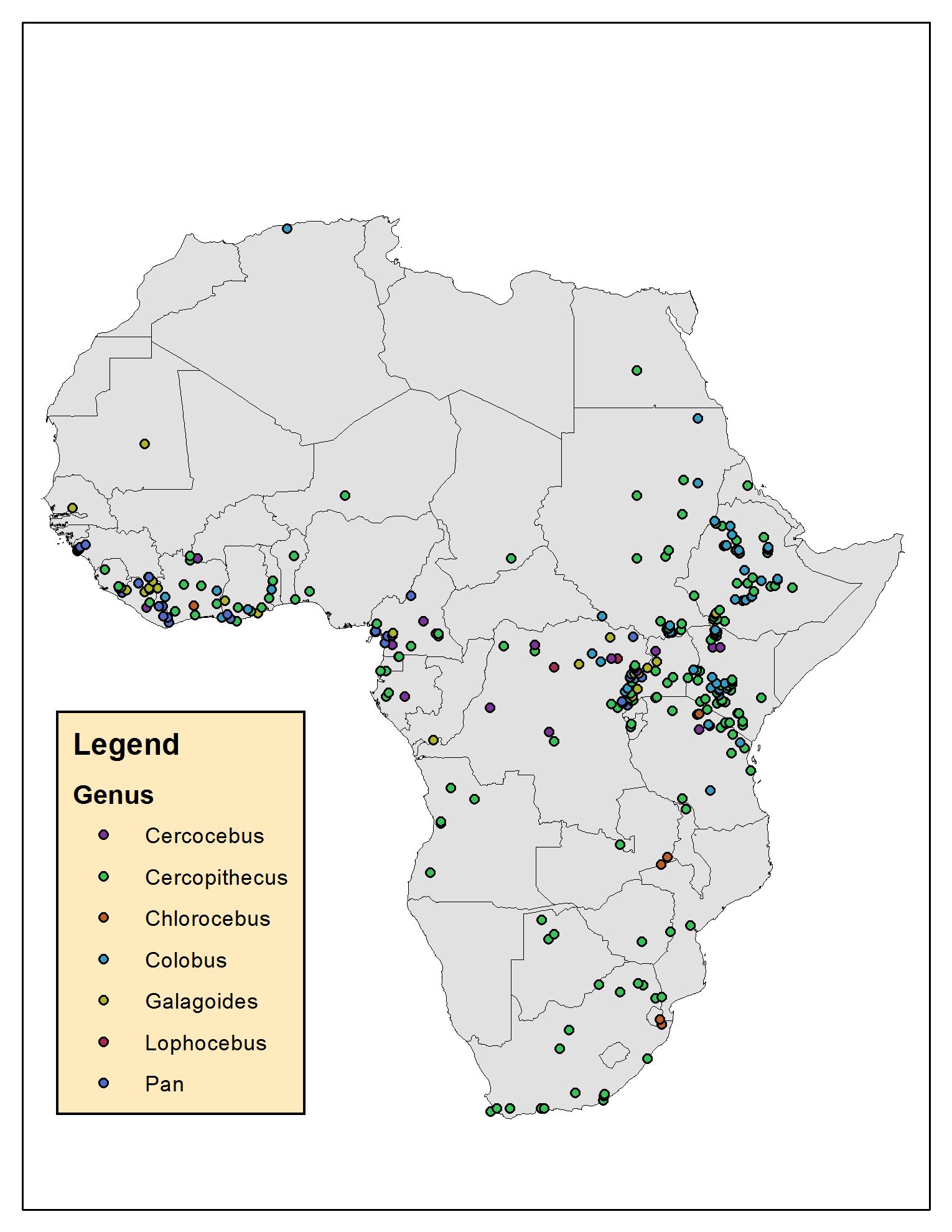

Supplement: Supplementary file 3 — Additional file 3: Coordinates for primate species records used in ENM modeling. Data from http://www.gbif.org was used to create ENMs for the 15 primate species. (JPEG 234 KB) [file 12942_2014_602_MOESM3_ESM.jpeg]
